# A review of policies on the involuntary use of psychotropic medications among persons experiencing incarceration in the United States

**DOI:** 10.1186/s40352-023-00204-1

**Published:** 2023-02-18

**Authors:** Joana Orta, Catherine Barton, Patricia Ilao, Dorie E. Apollonio

**Affiliations:** 1 School of Pharmacy, University of California. UCSF Clinical Sciences Box 1390, 530 Parnassus Avenue, Suite 366, San Francisco, CA 94143 USA; 2 Department of Clinical Pharmacy, University of California. UCSF Clinical Sciences Box 1390, 530 Parnassus Avenue, Suite 366, San Francisco, CA 94143 USA

**Keywords:** Antipsychotic, Corrections, Policy, Prisoners, Mental health, United States, Psychotropic

## Abstract

**Background:**

In *Harper v. Washington* (1990), the United States Supreme Court established the right of states to involuntary medicate incarcerated individuals in emergency situations without a court order. The extent to which states have implemented this in correctional facilities has not been well characterized. This exploratory qualitative study sought to identify state and federal corrections policies relating to involuntary psychotropic medication for individuals who are incarcerated and classify them by scope.

**Methods:**

State Department of Corrections (DOC) and Federal Bureau of Prisons (BOP) policies relating to mental health, health services, and security were collected between March and June 2021 and coded using Atlas.ti software. The primary outcome was whether states allowed emergency involuntary use of psychotropic medications; secondary outcomes pertained to use of restraint and “use of force” policies.

**Results:**

Of the 35 states plus the Federal BOP that made policies publicly available, 35 out of 36 (97%) allowed the involuntary use of psychotropic medications in emergency situations. The extent of detail contained in these policies varied, with 11 states providing minimal information to guide use. One state (3%) did not allow public review of “use of restraint” policies, and 7 states (19%) did not allow public review of “use of force” policies.

**Conclusions:**

More explicit criteria for emergency involuntary use of psychotropic medications are needed to better protect individuals who are incarcerated, and states should provide more transparency regarding use of restraint and use of force in corrections.

## Introduction

In the United States, individuals who are incarcerated can legally be medicated against their will. The legality of involuntary medication in emergency situations was established by the Supreme Court in *Washington v. Harper* (1990), wherein the due process clause was invoked to justify involuntary medication of inmates considered to be a danger to themselves or to others (Black, 2008). The court determined that the state’s interests could outweigh an individual’s liberty interest, and that in this event state interest allowed medication. After this decision, prison systems at the federal and state levels established policies in which they allowed involuntary medication of incarcerated people (Dlugacz & Wimmer, 2013). This tort law exemption has led to controversy surrounding what staff in correctional facilities deem to be an “emergency” and whether this exemption is invoked inappropriately in situations where staff attempt to control inmates; use of these medications has been referred to as “chemical restraints” (Auerhahn, 2000; Dlugacz & Wimmer, 2013).

There are a range of potential harms associated with involuntarily medicating people who are incarcerated. These include adverse effects associated with antipsychotics, such as extrapyramidal symptoms, sedation, weight gain, in addition to the trauma of being medicated against their will (Gross, 2002; Hervas et al., 2019). In states with nonexistent policies or policies that provide scant detail, there is room for interpretation of how to handle emergency situations that can increase the risk of medical errors. According to the Institute for Safe Medication Practices (2007), half of all medication errors that occur happen during the medication administration stage. Additionally, injectable medications, preferred by prison staff in emergency settings, are the cause for about two-thirds of those medication errors (Dlugacz & Wimmer, 2013; Institute for Safe Medication Practices, 2007). Equally important, involuntary medication is considered to be a violation of fundamental human rights to control one’s own body, and as a result, outside of correctional facilities, is undertaken only with significant legal protections (Black, 2008).

The potential scope of involuntary medication policies is substantial given that individuals with serious mental illness are overrepresented in U.S. jails and prisons (James et al., 2006; Prins, 2014). The National Institute of Mental Health (2021) defines serious mental illness as “a mental, behavioral, or emotional disorder resulting in serious functional impairment, which substantially interferes with or limits one or more major life activities.” As of 2019, the National Survey on Drug Use and Health estimated that 5.2% of adults living in the U.S. had a serious mental illness (National Institute of Mental Health, 2021). In comparison, 2012 data from the U.S. Department of Justice estimated the prevalence of serious mental illness to be 14% within state and federal prisons and 26% within local jails (Bronson et al., 2017). Previous estimates from 2000 to 2009 in U.S. prisons and jails are consistent with these data, with various studies concluding prevalence of serious mental illness in prison inmates to be around 15–20% (Torrey et al., 2010). These data are more striking when comparing numbers of individuals with serious mental illness at correctional facilities versus mental health facilities. Prisons have been deemed “the new asylums,” with one report by the Treatment Advocacy Center noting that state prisons and county jails hold 10 times the number of people with serious mental illness than state mental hospitals (Shenson et al., 1990; Torrey et al., 2010). Given that people who are incarcerated are a protected research class, the extent of mental illness among people in correctional facilities is not well characterized. Concerns about high levels of use and potential for misuse of drugs prescribed for prisoners with mental illnesses, notably antipsychotics, are warranted due to drug dependence being a major destabilizing factor in this population (Hervas et al., 2019).

Existing research has considered the legal aspects pertaining to involuntary medication, with particular interest in involuntary medication in the setting of restoring competency for trial (Gross, 2002). The study by Gross (2002) considered the legal framework within a single state, California, for forced antipsychotic use in state prisons. Other studies have considered health effects; for example, examining the effects of forced antipsychotic medication in nonemergency situations on prison inpatient days and disciplinary charges (Salem et al., 2015). To our knowledge, however, there have been no prior studies assessing the extent and nature of involuntary medication policies in prisons and jails in the U.S., despite their implications for the health of those who are incarcerated. As a result, the aims of this study were twofold: to identify state and federal corrections policies relating to involuntary psychotropic medication for individuals who are incarcerated and to classify these policies by scope.

## Methods

This qualitative study collected documents from two sources: state Department of Corrections (DOC) policies and Department of Justice Bureau of Prisons (BOP) policies for federal prisons. Researchers obtained state legal codes by accessing documents posted on the websites of state DOCs, state legislatures, through NexisUni government documents searches, and by making public records requests. BOP policies were downloaded from the Department of Justice website. Public records requests were reserved for situations in which there was either no relevant code found with other methods, or when publicly available codes relevant to the research were missing from the websites. Relevant codes included those that referenced involuntary medication, forced medication, or psychotropics, and policies addressing use of force, restraints, and consent. When available, the entirety of state codes categorized under mental health, health services, and security sections were downloaded from websites and saved.

In March–June 2021, three authors (redacted) collected state and federal policies regarding involuntary psychotropic use in U.S. federal and state prison systems. Responses to records requests were accepted until June 25, 2021, and any states that did not respond by this time were recorded as missing. All documents were uploaded to Atlas.ti for coding analysis using a shared remote project location. A keyword search using the following terms: involuntary, force, restraint, mental, consent, psych, medication, antipsychotic, psychotropic, emergency, commitment, and suicide was performed to scan through the documents. A list of codes was assigned to the text (Table 1).

**Table 1:** Initial code list assigned to documents using Atlas.ti

commitment	criteria	hospitalization	policy	self-harm
competency	DOT	involuntary	psychotropic	suicide
compliance	duration	KOP	restraint	voluntary
consent	emergency	mental health	rights	
covid	force	non-emergency	seclusion	

*DOT* duration of therapy, *KOP* keep on person

Following the initial keyword search and coding, relevant policies were read in full. Initially, three sets of policies (Alaska, Arizona, Federal BOP) were triple coded by each author, and disagreements in coding were discussed together with a fourth reviewer (redacted) who made a final determination in the event that the group could not reach consensus. After this first round of coding, three additional sets of policies (Iowa, Wisconsin, Wyoming) were double coded by two authors each to validate the coding strategy; at that point there were no further disagreements. After validating the coding strategy for these six states, the authors coded the remaining states individually.

The primary outcome assessed was whether states allowed involuntary use of psychotropic medication in emergency situations without a court order. Secondary outcomes included whether policies made note of involuntary medication with a court order, had a restraint policy, or had a use of force policy. Policies on involuntary administration of psychotropic medications were further characterized using quotations that illustrated key points and were categorized based on the extent of information provided. To analyze the comprehensiveness of the involuntary medication policies enforced by each state, we categorized policies as minimal, moderate, or extensive based on a 6-point scoring system. One point was assigned for each of the following six details outlined in policy text: (a) definitions of key terms, (b) specific duration of treatment allowed, (c) less restrictive measures to be attempted prior to involuntary medication administration, (d) post-administration monitoring, (e) documentation requirements, and (f) references to *Harper* (1990) or other state and federal policies. A policy scoring 1–2 points was deemed minimal, 3–4 points moderate, and 5–6 points extensive.

We estimated the number of individuals potentially affected by these policies, both in total and as a share of state population, by downloading 2019 data from The Sentencing Project (2020).

## Results

### Policy availability

In total, we were able to obtain policies on involuntary administration of psychotropic agents for 40 states and the U.S. Federal Bureau of Prisons. Records for Louisiana, New York, Texas, and Wisconsin were not posted publicly but were successfully obtained by making a records request.

Eleven states had missing or incomplete data (Florida, Mississippi, Missouri, Nebraska, North Dakota, Rhode Island, South Carolina, Tennessee, Utah, West Virginia, and Virginia). These states either provided no new relevant documents after a public records request (South Carolina), required a research proposal and IRB review (Tennessee), determined that the researchers did not meet disclosure requirements, typically residency in that state (Utah, Virginia, West Virginia), or did not respond to the request (Mississippi, Missouri, Nebraska, North Dakota, Rhode Island). A search for Florida state documents only identified policies that at the time of the search had been either repealed or withdrawn.

Although records were obtained for Connecticut, Maine, and Michigan, policies on involuntary medication administration were unclear and did not differentiate explicitly between emergency versus non-emergency situations. North Carolina’s policy was only applicable to a subset of its incarcerated population, specifically those housed and treated in inpatient or residential mental health programs. Given the insufficient data, lack of clarity, and limited applicability of these policies, the decision made after coding was to exclude the data from these 16 states in our analysis.

Emergency vs. non-emergency use of involuntary medication administration.

**Fig. 1:**
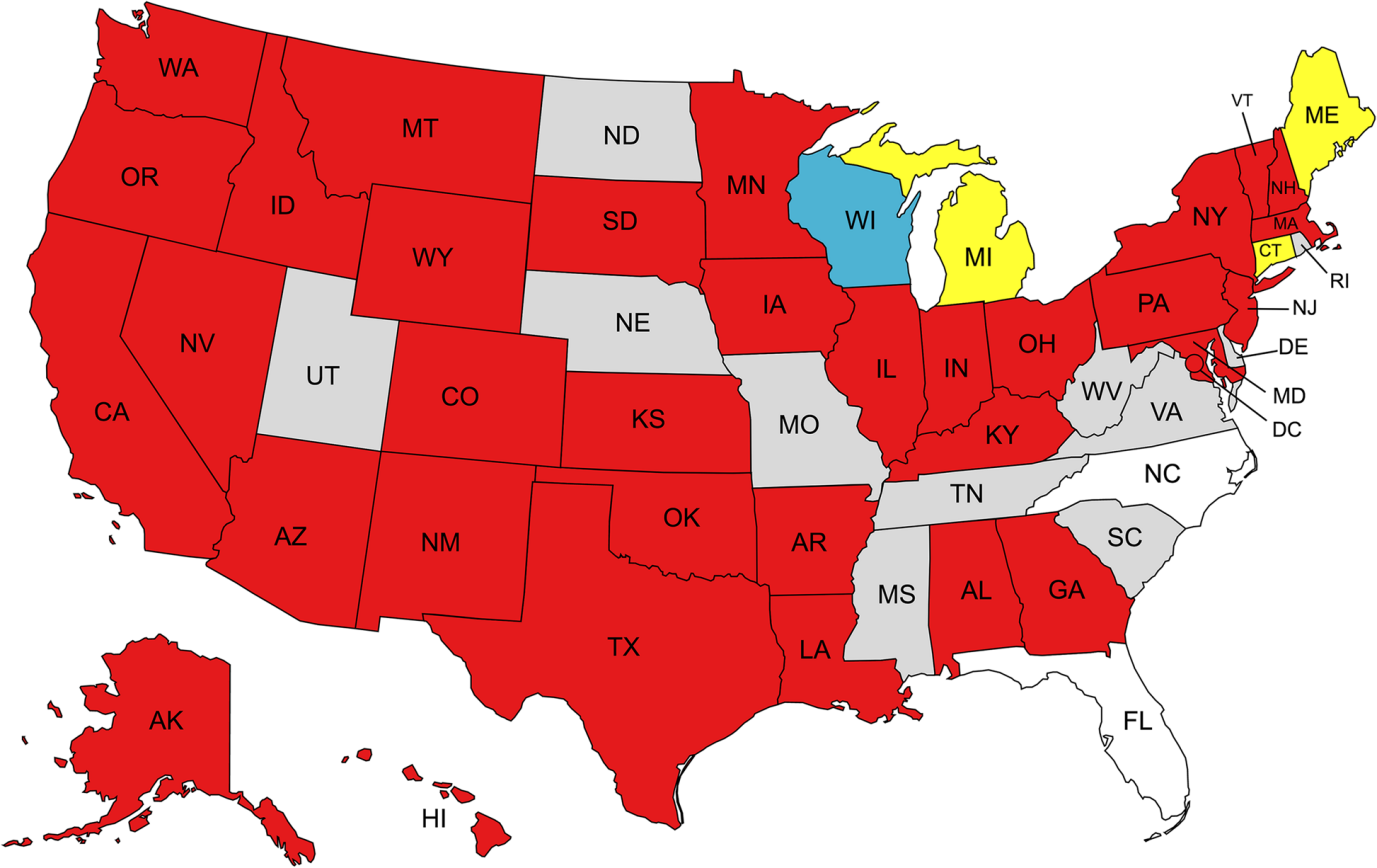
Policies regarding emergency and non-emergency involuntary administration of psychotropic medication across the United States. Federal Bureau of Prisons is represented by District of Columbia (DC). Red = available policies on involuntary administration of psychotropic medication explicitly allow use in emergency situations, Blue = available policies on involuntary administration of psychotropic medication allow use in non-emergency situations, but do not specify use in emergency situations, Yellow = available policies on involuntary administration of psychotropic medication do not distinguish between emergency and non-emergency use, Gray = no policy data or insufficient data, White = excluded from analysis due to withdrawn policy or limited in scope. Source: Data collected by the authors; map created in mapchart.net

**Fig. 2:**
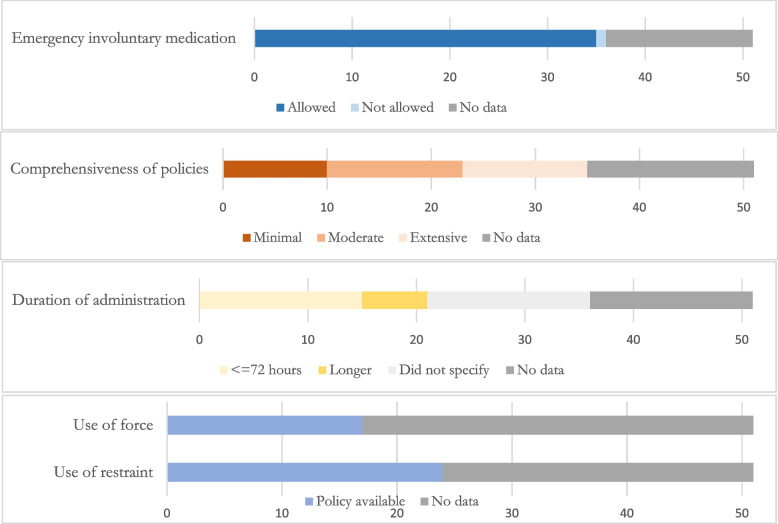
Summary of policy characteristics. Source: Data collected by the authors

Of the 35 states and the U.S. Federal Bureau of Prisons that made their policies available and were included in analysis, 35 out of 36 (97%) explicitly allowed the emergency involuntary administration of psychotropic medications to individuals who are incarcerated without a court order, pursuant to *Harper v. Washington* (1990) (Figs. 1, 2). According to 2019 prison population estimates gathered from The Sentencing Project (2020), these policies cover over 1 million people in the United States and 76% of the total U.S. prison population. Most states had policies detailing emergency as well as non-emergency situations for which they allowed involuntary medication administration; unlike in emergency situations, non-emergency situations require a hearing process prior to administration. Wisconsin was the only state with clear, available policies that solely described use in non-emergency situations.

### Comprehensiveness of policies

We categorized the comprehensiveness of information provided in policies on emergency use of involuntary psychotropic medications; as described in methods, some states indicated that they defined key terms to establish what constituted appropriate use, limited the duration of treatment allowed, required less restrictive measures to be attempted prior to involuntary medication administration, required monitoring post-administration, required documentation of involuntary medication, or made references to *Harper* (1990) or other state and federal policies. Overall, 10 states plus the BOP (31%) provided minimal information on their policies (1–2 of 6 potential inclusions), 13 states (36%) provided “moderate” information (3, 4), and 12 (33%) states provided “extensive” information (5, 6) (Fig. 2). Variation in the content of state policies is illustrated by sample quotations in Table 2. Wisconsin did not have an emergency administration policy and was not included in our evaluation of comprehensiveness.

**Table 2:** Sample quotations from the text of policies on emergency involuntary administration of psychotropic medications

State	Policy text
Alaska	“Emergency Forced Medication: Inmates have the right to refuse psychotropic medication except in psychiatric emergencies or when administrative procedures have been conducted consistent with the Supreme Court Washington v. Harper decision. Psychiatric emergencies occur when an inmate poses an imminent threat to self or others and all less intrusive measures have been attempted or judged by the psychiatrist to be inadequate.” “Involuntary Medication: A legal process for the administration of psychotropic medication to an inmate who refuses voluntary treatment.”
Massachusetts	“The [emergency] involuntary administration of psychotropic medication may be used if: a. An inmate poses a clear and immediate threat to harm him/herself or others; or to prevent the immediate, substantial and irreversible deterioration of a serious mental illness of an inmate who is currently incapable of making informed medical decisions on their own behalf; and b. All less restrictive or intrusive measures have been employed or have been judged by the treating psychiatrist, on-call psychiatrist, or physician to be inadequate. Authorization for involuntary administration of psychotropic medication that is specifically limited to a single dose of such medication.”
South Dakota	“In an emergency, involuntary treatment of an inmate with psychotropic medication may be administered without panel review for up to 10 days if the treatment is ordered by two physicians, one of whom shall be a psychiatrist.”

Policies pertaining to the emergency use of involuntary psychotropic medications often included the set of criteria laid out by *Harper* that constituted emergency use situations. This was generally described as “if the prisoner suffered from a mental disorder and was gravely disabled or posed a likelihood of serious harm to himself, others, or their property.” Six states (Alabama, Arkansas, Colorado, Georgia, Idaho and Montana) made reference directly to *Harper* or more generally to laws and policies of the jurisdiction. A total of 23 states plus the BOP added that less restrictive measures must be attempted prior to use, compared to 11 states that made no specification.

Twenty-one policies (20 states and the BOP) specified a duration of allowed administration. Of those 21 policies, 15 states and the BOP (76%) allowed up to 72 hours, and 6 states (29%) allowed greater durations (Fig. 2). These greater durations included 96 hours (Massachusetts), 7 days (Georgia), 10 days (Colorado, South Dakota), and up to 14 days prior to administrative review (Minnesota, Montana). Some states did not specify duration, instead stating that the physician or psychiatrist would specify duration of treatment (Louisiana, New Mexico, Nevada), or specified a limited number of doses to minimize harm prior to transfer to a psychiatric facility (New York). Louisiana placed no limit on duration for youths under 18, stating that involuntary psychotropic medication administration could take place “as long as the emergency continues.”

Georgia was the only state in which the involuntary psychotropic administration policy indicated what types of medications were allowed during emergency situations. The policy outlined that only short-acting injectable medications could be used during emergency situations and that medications such as haloperidol decanoate, which can remain in the system for weeks, could not be administered.

### Use of restraint and use of force policies

Out of the 35 states and the Federal BOP included in the primary outcome analysis, a total of 24 states and the BOP (69%) made their use of restraint policies available (Fig. 2). Full access to these policies was explicitly restricted only by Kentucky; policies were referenced in other documents but not made available or restricted in two states (South Dakota, Wyoming). The remaining 17 states (47%) made no mention of any policy.

Use of force policies were made available by 17 states (47%) plus the BOP (Fig. 2). A total of 7 states (19%) restricted access to these policies (Arizona, Colorado, Hawaii, Kentucky, New York, South Dakota, Vermont). Five states (14%; Maryland, New Mexico, Washington, Wisconsin, Wyoming) referenced an existing use of force policy that was not publicly available or explicitly restricted. The remaining 14 states (39%) made no mention of such policies.

## Discussion

Policies that govern correctional facilities in the US have the potential to impact over a million people; our findings indicate that the overwhelming majority of states that allowed public review of their policies permit individuals who are incarcerated to be medicated against their will. As a result, it is imperative to understand these policies, their potential impacts, potential discrepancies in treatment of vulnerable persons. Overall, only 41 of the 50 states and the US Bureau of Prisons made their policies accessible. Of these 41, 36 (88%) provided public access to their policies regarding involuntary administration of psychotropic medications in emergency situations and 35 states permitted it. In these situations, without a court hearing to evaluate the need for medication and without an appeals process, people who are incarcerated lose autonomy and decisions about their best interest may rest with people who are not mental health professionals. People who are incarcerated are also physically separated from family and community members who could act as advocates for their treatment.

There are persistent concerns when considering involuntary medication that emergency situations can be loosely defined, allowing staff to use involuntary medication as “chemical restraints” (Auerhahn, 2000; Dlugacz & Wimmer, 2013). In addition to these intended effects, which may not be consistent with the desires of the individual being medicated, antipsychotics have multiple short- and long-term adverse effects and unclear guidelines about administration increase the risk of these harms, as well as increasing the risk of medication errors. Outside of correctional facilities, these considerations have generated strong legal protections that limit administration of involuntary medication. However within correctional facilities, the BOP and states provided little detail about involuntary medication policies that increase risks to people who are incarcerated. For example, 20 states specified the allowed duration for administering involuntary medication while 14 others and the BOP did not. Minimal or vague policies have the potential to create confusion among staff who may be unclear as to what qualifies an event as an emergency and their appropriate role in the situation. A staff member who administers psychotropic medication to an inmate involuntarily during a deemed emergency may also be able to use restraints, chemical weapons, and other methods of force during the encounter. Having clearly defined policies that indicate what steps are appropriate to ensure the safety of the staff and those who are incarcerated, establish responsibility, and ensure accountability would benefit both people at risk of involuntary medication and staff in correctional facilities, and reduce medical errors. Staff at correctional facilities that do not have detailed policies may not be able to adhere to intended rules surrounding emergency use of involuntary medication, potentially infringing further on the rights of those incarcerated and leading to inappropriate medication administration.

We also found that many states did not allow review of their policies, raising concerns regarding transparency. Access was most restricted for use of force policies. It is unclear why special records requests were required for policies in 14 states and why one state listed a repealed policy, given that 35 states and the BOP made their policies and procedures manuals fully accessible. If a state does not make their policy available, it is impossible to assess whether they are following the guidelines set forth by *Harper v. Washington* (1990) and as a result, individuals covered by these policies may be subject to practices that overstep legal boundaries. These risks are borne disproportionately by people with serious mental illness, who are overrepresented in the incarcerated population. The overlapping vulnerabilities of people who are incarcerated and people with serious mental illness suggest a greater need for transparency and protection when creating policies guiding both involuntary medication and use of restraints and force. However existing policies in most states regarding use of restraints and force are unavailable for review.

Our study has limitations. As noted above we were unable to locate relevant, explicit, or current policies for 15 states, resulting in an incomplete sample. Nonetheless our sample included a majority of states as well as policies for federal prisons, which account for 64% (state) and 12% (federal) of the population of people incarcerated in the U.S., respectively (The Sentencing Project, 2020). We also assumed that the policies listed by each state were enforced as written and did not attempt to validate current practices; it is possible that certain states changed practices without updating their policies. Finally, it is unclear how policies are actually implemented, and we did not review policies pertaining to staff to assess whether there were consequences for violations. Nonetheless, these findings provide new insight into the scope of involuntary medication policies that expand on existing research assessing the health effects of forced antipsychotic medication (Salem et al., 2015). Potential avenues for future research could include site studies at different correctional facilities to assess how policies have been implemented and enforced, as well as long-term follow-up of people who have been involuntarily medicated during incarceration to assess potential related health outcomes.

## Conclusions

This study identified a need for greater transparency regarding policies that guide treatment of individuals who are incarcerated. It also demonstrated that individuals may be subject to more or less comprehensive policies that guide administration of involuntary psychotropic medication depending on the state in which they are incarcerated, a situation that compromises equity and justice. These differences in policies may also result in inappropriate use if protections and documentation are not in place. Greater consistency across states with respect to involuntary medication policies, particularly for states that have provided little or no information for public reivew, is warranted. For the protection of both people who are incarcerated and staff in correctional facilities, states should provide clear guidelines regarding the duration of allowed administration, the types of medications that may be used (i.e., short-acting over long-acting antipsychotics), outlined procedures for documentation, and follow-up following use of emergency psychotropic medications. Without this information, it is impossible to assess whether incarcerated people are provided appropriate legal protections, or to protect their mental and physical health.

## Data Availability

The data used in this study are available in the public domain. The aggregated dataset analyzed for the current study is available at 10.7272/Q6KD1W4D

## References

[CR1] Auerhahn K, Leonard ED (2000). Docile bodies? Chemical restraints and the female inmate. The Journal of criminal law & criminology.

[CR2] Black, L. (2008). Forced medication of prison inmates. *The virtual mentor : VM*, *10*(2), 106–109. 10.1001/virtualmentor.2008.10.2.hlaw1-0802. 10.1001/virtualmentor.2008.10.2.hlaw1-0802 23206823

[CR3] Bronson, J. & Berzofsky, M (2017). Special Report: Indicators of Mental Health Problems Reported by Prisoners and Jail Inmates, 2011-12. *Bureau of Justice Statistics*. US Department of Justice Office of Justice Programs.

[CR4] Dlugacz H, Wimmer C (2013). Legal aspects of administrating antipsychotic medications to jail and prison inmates. International Journal of Law and Psychiatry.

[CR5] Gross DE (2002). Presumed dangerous: California's selective policy of forcibly medicating state prisoners with antipsychotic drugs. University of California, Davis law review.

[CR6] Hervás G, Ruano C, Sanz-Alfayate G, Algora I, Celdran MA, Mur MA (2019). Analysis of the management of antipsychotics in a group of prisons. Revista espanola de sanidad penitenciaria.

[CR7] Institute for Safe Medication Practices (ISMP). (2007). Errors with Injectable Medications: Unlabeled Syringes are Surprisingly Common. https://www.ismp.org/resources/errors-injectable-medications-unlabeled-syringes-are-surprisingly-common. Accessed 17 Jan 2023.

[CR8] James, D. J., & Lauren E. G. (2006). Special Report: Mental Health Problems and Prison and Jail Inmates. US Department of Justice Office of Justice Programs. *Bureau of Justice Statistics*.

[CR9] Prins, S. J. (2014). Prevalence of mental illnesses in US State prisons: a systematic review. *Psychiatr Serv 65*(7):862–72. 10.1176/appi.ps.201300166. 10.1176/appi.ps.201300166 PMC4182175 24686574

[CR10] Salem A, Kushnier A, Dorio N, Reeves R (2015). Nonemergency involuntary antipsychotic medication in prison: Effects on prison inpatient days and disciplinary charges. The Journal of the American Academy of Psychiatry and the Law.

[CR11] Shenson D, Dubler N, Michaels D (1990). Jails and prisons: The new asylums?. American Journal of Public Health.

[CR12] The Sentencing Project. U.S. Criminal Justice Data [dataset]. (2020). https://www.sentencingproject.org/research/us-criminal-justice-data/. Last updated 2023. Accessed 17 Jan 2023.

[CR13] Torrey EF, Kennard AD, Eslinger D, Lamb R, Pavie J (2010). More mentally ill persons are in jails and prisons than hospitals: A survey of the states.

[CR14] US Department of Health and Human Services (US DHHS), National Institute of Mental Health (NIMH). (2021). Mental Illness. https://www.nimh.nih.gov/health/statistics/mental-illness. Last updated January 2022. Accessed 17 Jan 2023.

